# Dissemination of *bla*_NDM_-harboring plasmids in carbapenem-resistant and hypervirulent *Klebsiella pneumoniae*

**DOI:** 10.1128/spectrum.01968-24

**Published:** 2025-02-12

**Authors:** Yihai Gu, Xiao Wang, Wei Zhang, Rui Weng, Qiucheng Shi, Xuan Hou, Hui Wang, Minghui Deng, Jian Mou, Yan Jiang

**Affiliations:** 1 Department of Microbiology, School of Medicine, Xi’an Jiaotong University, Hanzhong, China; 2 Department of Laboratory Medicine, Chengdu 363 Hospital Affiliated to Southwest Medical University, Chengdu, China; 3 Department of Infectious Diseases, Sir Run Run Shaw Hospital, Zhejiang University School of Medicine, Hangzhou, China; University of Guelph College of Biological Science, Guelph, Ontario, Canada

**Keywords:** carbapenem-resistant *Klebsiella pneumoniae*, *bla*_NDM_-harboring plasmids, molecular epidemiological, hypervirulent

## Abstract

Carbapenem-resistant *Klebsiella pneumoniae* (CRKP) is a global public health threat, and CRKP strains carrying *bla*_NDM_ plasmids are of particular concern. Here, the antimicrobial resistance and molecular epidemiological characteristics of 59 CRKP strains isolated from patients receiving care at a tertiary hospital in China from 2019 to 2021 were determined through antimicrobial resistance testing and genomic sequence analysis. We analyzed the genetic characteristics and transmissibility of *bla*_NDM_-harboring plasmids through S1-digested pulsed field gel electrophoresis, Southern blotting, and conjugation assays. The virulence levels of the strains were evaluated using a *Galleria mellonella* larvae infection model. All the CRKP strains were multidrug resistant, but none were resistant to colistin or tigecycline. A total of eight sequence types (STs) were detected in these CRKPs, with ST11 (39/59) and ST15 (12/59) being the most common. Among the carbapenemase genes, *bla*_KPC_ (39/59) and *bla*_NDM_ (18/59) were the most common, with *bla*_KPC-2_ transmitted mainly through ST11 clones and *bla*_NDM-5_ transmitted through ST15 clones. The *bla*_NDM_-harboring plasmids primarily belong to the incompatibility type IncX3 (14/18). IS*26*-mediated gene recombination, sequence insertion, and plasmid integration were observed to occur during the dissemination process. The resistance gene profile mediated by *bla*_NDM_-harboring plasmids can be transferred through conjugation, which have almost no fitness cost and is extremely stable after passage. Six of these strains presented carbapenem resistance combined with hypervirulence (CR-hvKP), including ST23-KL1, ST218-KL57, ST17-KL112, and ST147-KL64. Additionally, *bla*_NDM_-harboring plasmids can disseminate rapidly over large surface areas and persist for a long time in medical institutions, leading to outbreaks of highly resistant clones associated with CR-hvKP.

**IMPORTANCE:**

The appearance of plasmids carrying *bla*_NDM_ and the rise of carbapenem-resistant hypervirulent *Klebsiella pneumoniae* (CR-hvKP) have significantly escalated the public health risk associated with Carbapenem-resistant *Klebsiella pneumoniae* (CRKP). In this study, we described the resistance rates, virulence factors, and molecular epidemiology of 59 CRKP isolated from patients at a tertiary hospital in China from 2019 to 2021. Beyond this, we also assessed the characteristics of *bla*_NDM_-harboring plasmids and their dissemination patterns among CRKP, especially some CR-hvKP. Our research contributes to the field of *bla*_NDM_ dissemination studies, prompting researchers to formulate strict measures to prevent and control the development of *bla*_NDM_-harboring plasmids, which is instrumental in the clinical development of targeted treatment plans.

## INTRODUCTION

In recent years, the global incidence rate of carbapenem-resistant *Klebsiella pneumoniae* (CRKP) infection has substantially increased (1), and the treatment of CRKP infection is extremely challenging. Many studies have shown that infection with CRKP can lead to increased mortality in patients (2–4), which poses a major threat to public health. In addition to its complexity, the emergence of carbapenem-resistant hypervirulent *Klebsiella pneumoniae* (CR-hvKP) presents a formidable challenge characterized by its dual traits of heightened virulence and resistance. Infection with CR-hvKP strains often results in poor patient outcomes.

CRKP produces carbapenemases, the most common of which is KPC (a class A serine β-lactamase) followed by NDM (a class B metallo β-lactamase) and OXA-48 (a class D serine β-lactamase), and these enzymes are responsible for CRKP resistance to most β-lactamase drugs, including carbapenem. The CRKP strain that produces KPC has been widely identified, and ST11-*bla*_KPC-2_ is the most dominant genotype of CRKP (5, 6). The number of NDM-producing CRKP strains is relatively small, and ST11, 290, 147, 340, 15, and 278 are common sequence types (STs) in NDM-producing CRKP strains worldwide (7). The NDM-producing CRKP strains have a high diversity of STs, and high-risk clones producing NDM are relatively rare (8–10). To our knowledge, there have been reports of NDM-producing CR-hvKP isolates from a single ST23 isolate in Saudi Arabia, the UK, and China (11–13), one ST11 and one ST15 isolate in India (14), one ST14 isolate and one ST86 isolate in China (15, 16), three isolates from South India (17, 18), and seven ST1764 isolates in China (19). NDM was first discovered in *K. pneumoniae* in 2008, and since then, NDM-producing *K. pneumoniae* strains have been detected worldwide. NDM-1 and NDM-5 are the most popular, and there are only two amino acid substitution differences between them (20). NDM can hydrolyze most β-lactam drugs, including carbapenem, and cannot be inactivated by new β-lactamase inhibitors, such as avibactam and farborbactam (21). Moreover, most *bla*_NDM_-harboring plasmids are disseminated (22) widely in *Enterobacteriaceae*, and studies have shown that the wide spread of conjugated *bla*_NDM_-harboring plasmids has accelerated the emergence and prevalence of carbapenem-resistant hypervirulent *K. pneumoniae* (CR-hvKP) (23), which is a great threat to public health.

In this study, we characterized the molecular features of CRKP strains isolated from patients receiving care at a tertiary hospital in China from 2019 to 2021. Given that the characteristics of KPC carbapenemase-producing strains have been extensively reported previously, we focused on the dissemination of *bla*_NDM_-harboring plasmids, particularly in some CR-hvKP strains.

## MATERIALS AND METHODS

### Strains

We retrospectively analyzed 59 CRKP isolates from patients receiving care at Hanzhong 3201 Hospital, Shaanxi Province, China from January 2019 to December 2021. Only the first CRKP isolate cultured from each patient was used as the subject of our research, ensuring that there were no duplicate strains included in our analysis. CRKP was defined as intermediate or resistant strains that were present after treatment with any carbapenem antibiotic (meropenem, imipenem, or ertapenem).

### Antimicrobial susceptibility testing

The minimal inhibitory concentrations (MICs) of 16 antimicrobial agents were tested via broth microdilution. *Klebsiella pneumoniae* ATCC 700603 and *Escherichia coli* ATCC 25922 were used for quality control. Clinical breakpoints of antibiotics were interpreted according to the Clinical and Laboratory Standards Institute M100 guidelines (24), except for tigecycline, which was interpreted according to the US Food & Drug Administration criteria (25).

### Whole-genome sequencing and sequence analysis

Bacterial DNA was extracted with a QIAamp DNA Purification Mini Kit (QIAGEN GmbH, USA) and sent for next-generation sequencing on the Illumina HiSeq platform (Illumina, San Diego, USA). After the raw sequence data were obtained, the Shovill pipeline (https://github.com/tseemann/shovill) was used to filter and assemble the whole-genome sequence. In accordance with the ST of the strain and the estimated size of the *bla*_NDM_-harboring plasmid, seven representative strains (KP1930, KP2050, KP2058, KP2062, KP2063, KP2068, KP2174, and KP2190) were selected for long-read sequencing on MinION platforms (Nanopore, Oxford, UK), which were hybrid assembled by using Unicycler (v.0.4.8) (26).

Core genome multilocus sequence typing (cgMLST) analysis of isolates was performed by comparing the 2,358 core genes of *K. pneumoniae* using the software Ridom SeqSphere+ (v.4.1.9) (Ridom GmbH, Germany), with which a phylogenetic tree was constructed. If the number of different core genes between each pair of strains was ≤7, they were considered homologous clones. Kleborate (v.2.2.0) was used to screen genome assemblies for MLST, antimicrobial resistance genes, capsular type, and virulence loci such as siderophore systems *yersiniabactin* (*ybt*), *aerobactin* (*iuc*), *colibactin* (*clb*), and *salmochelin* (*iro*), and the hypermucoid locus *rmpADC*/*rmpA2* (27). Sequence comparisons were performed and visualized using the Proksee online tool (https://proksee.ca) and Easyfig (v.2.2.5) software.

### S1-digested pulsed field gel electrophoresis (S1-PFGE) and Southern blot hybridization analysis

S1-PFGE was performed using the contour-clamped homogeneous electric field technique as described previously (28). Briefly, genomic DNA was digested with S1 nuclease (Takara, Dalian, China), and electrophoresis was performed at 14°C and 6 V/cm in a 2.16–63.8 s pulse time gradient with an alternating pulse at 120°C for 17 h for 30 min. The DNA fragment digested with S1 nuclease was subsequently transferred to a positively charged nylon membrane. Southern blotting was performed via the DIG High Prime DNA Labelling and Detection Kit (Roche, Basel, Switzerland). The DNA probe was a *bla*_NDM_ fragment amplified by PCR using specific primers (NDM_F: 5′- actttggcccgctcaaggta -3′, NDM_R: 5′- cgggccgtatgagtgattgcg -3′).

### Conjugation assay

Eighteen NDM-producing isolates were subjected to conjugation experiments to determine the horizontal transfer of *bla*_NDM_-harboring plasmids. Using *E. coli* EC600 as the recipient bacteria, conjugates were screened on Mueller-Hinton (MH) agar-coated plates supplemented with ertapenem (1 µg/mL) and rifampicin (700 µg/mL). In addition, two NDM-producing isolates were resistant to rifampicin (KP1930, KP2058), so *E. coli* J53 was used as the recipient strain to screen the conjugate on MH agar-coated plates supplemented with ertapenem (2 µg/mL) and sodium azide (150 µg/mL). The strains identified as *E. coli* by mass spectrometry and the *bla*_NDM-_harboring plasmids amplified by PCR using specific primers were confirmed to be conjugates (NDM_F: 5′-atattatgcacccggtcgcga-3′, NDM_R: 5′-gcagcttgtcggccatgcg-3′).

### Plasmid stability assay

As reported previously (21), the stability of the *bla*_NDM_-harboring plasmid was analyzed. In brief, 18 conjugates were grown overnight (37°C, 200 rpm) and passaged continuously for 12 days after a 1:200 dilution in basal lysogeny broth (LB) without antibiotics. The culture was seeded into antibiotic-free MH agar-coated plates every 2 days, and 50 single colonies from each conjugate were selected from the plates. PCR was performed to confirm that the *bla*_NDM_-harboring plasmid was still present in the conjugates, and the *bla*_NDM_-harboring plasmids identified by specific amplification primers were subjected to a conjugation assay. All of the assays were repeated three times. Stability is expressed as the ratio of the number of single colonies with *bla*_NDM_ plasmids to 50.

### Relative growth rate determination

The growth rates of 18 CRKP strains producing NDM, the conjugates, and the recipient strains EC600 and J53 were determined as previously described (20). Three independent cultures of the subjects were grown overnight and diluted to 1:100 in LB, after which a 200 µL aliquot was placed into a flat-bottomed 100-well plate in triplicate. The plate was incubated at 37°C. The OD_600_ of each culture was measured every 5 min for 20 h via a Bioscreen C MBR machine (Oy Growth Curves Ab Ltd., Finland). Relative growth rates were estimated from the OD_600_ curves using an R script. The relative growth rate results were analyzed via one-way analysis of variance in GraphPad Prism 8. A *P*-value <0.05 was considered significant.

### Virulence evaluation in a *Galleria mellonella* larvae infection model

To evaluate the virulence of NDM-producing isolates, we selected seven representative strains according to different STs to construct a *G. mellonella* infection model. The *G. mellonella* infection model was established as described previously (28). Briefly, exponential growth phase bacterial cultures (37°C, 200 rpm, 4 h) were washed with sterile phosphate-buffered saline (PBS) and then adjusted to an OD_600_ of 1.0 /mL. The bacterial suspension was subsequently adjusted to a standardized concentration of 1 × 10^6^ colony-forming unit (CFU)/mL to ensure experimental consistency. Using the *G. mellonella* infection model, we inoculated each larva by injecting precisely 10 µL of the prepared bacterial suspension into the first left prolegs, with a sample size of 10 larvae per experimental group. A total of 10 larvae injected with 10 µl of PBS served as a combined trauma and solvent control. The samples were incubated at 37°C in the dark. The experiment was repeated three times per group for 72 h. The number of surviving larvae was counted daily, and a log-rank test was performed using GraphPad Prism 8.

## RESULTS

### Specimen types and clinical characteristics of patients with CRKP

Among the 59 CRKP isolates, 24 were derived from respiratory samples, accounting for 40.7% of the total number of samples, 14 and 7 were derived from blood and urine samples, accounting for 23.7% (14/59) and 11.9% (7/59) of the total number of samples, respectively, and six were derived from secretion samples, accounting for 10.2% of the total number of samples (6/59) (Table S1). The clinical information revealed that 57.6% (34/59) of the patients with CRKP infection were male and that 42.4% (25/59) were female, with an average age of 55.16 ± 19.79 years. The CRKP isolates were mainly from patients in the Intensive Care Unit (ICU) (30.5%, 18/59), followed by patients in the Department of Neurosurgery (20.3%, 12/59) and patients in the Department of Respiratory and Critical Care Medicine (11.9%, 7/59). In this study, no isolates were obtained from the pediatrics department (Table S2).

### Antimicrobial resistance of CRKP isolates

**Fig 1:**
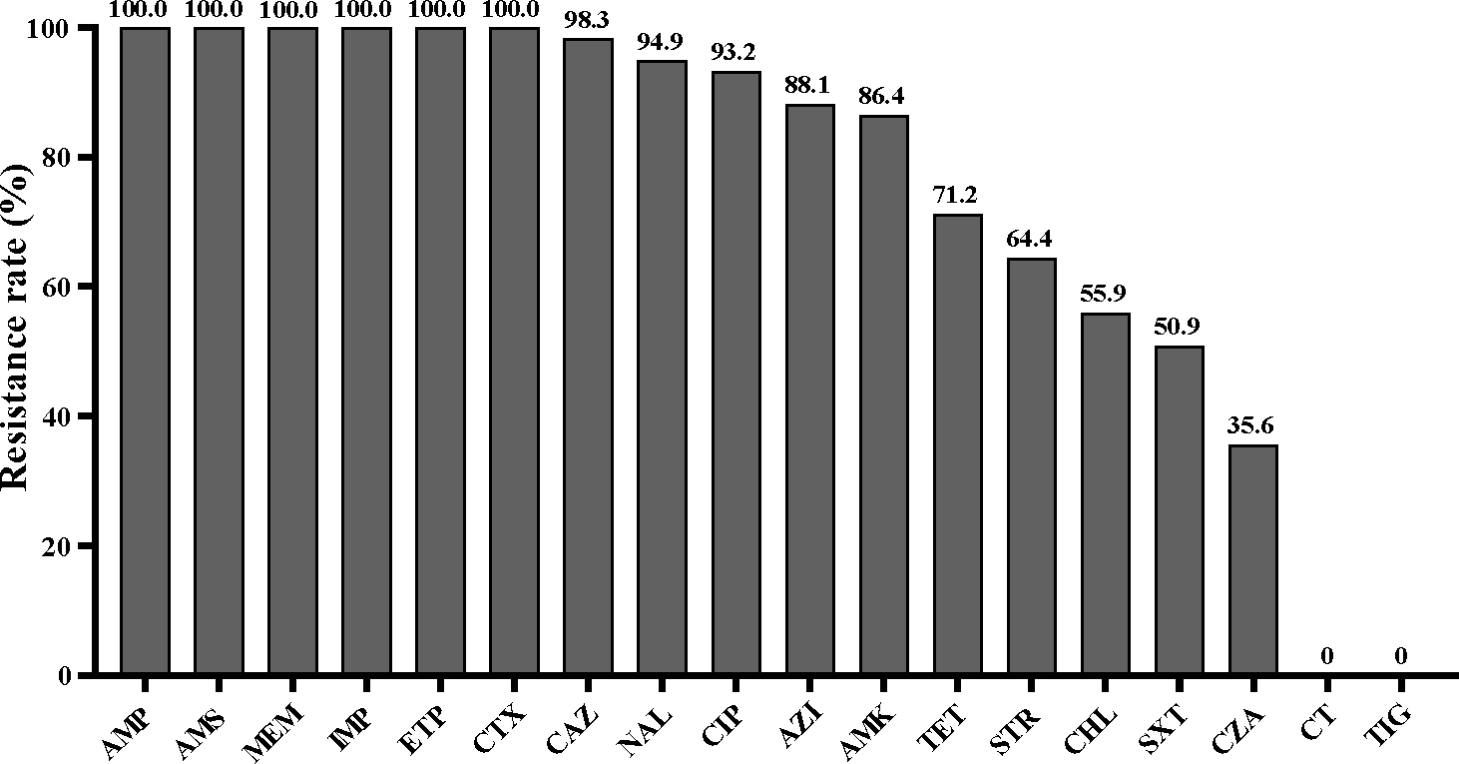
Results of antimicrobial susceptibility testing of 59 CRKP. MEM, meropenem; IPM, imipenem; ETP, ertapenem; CTX, cefotaxime; CAZ, ceftazidime; NAL, nalidixic acid; CIP, ciprofloxacin; AMK, amikacin; STR, streptomycin; AZI, azithromycin; TET, tetracycline; CHL, chloramphenicol; SXT, trimethoprim-sulfamethoxazole; CZA, ceftazidime-avibactam; CT, colistin; TIG, tigecycline.

The resistance rates of the CRKP isolates to carbapenems (meropenem, imipenem, and ertapenem) and cephalosporin (cefotaxime) were as high as 100%. The resistance rates to both ceftazidime and quinolones (nalidixic acid and ciprofloxacin) exceeded 90%. Notably, the resistance rates to ceftazidime/avibactam were as high as 35.6%. However, all CRKP strains were sensitive to colistin and tigecycline (Fig. 1).

### Genetic characteristics of CRKP

Fifty-nine CRKPs were divided into eight STs (Table 1), including ST11 (66.1%, 39/59) and ST15 (20.3%, 12/59). Notably, two isolates belonging to ST23, a typical clone of hvKP, were identified. Among the 59 isolates, the acquired carbapenemase genes included *bla*_KPC-2_ (66.1%, 39/59), *bla*_NDM_ (30.5%, 18/59), and *bla*_IMP-4_ (1.7%, 1/59) (Table 1). A total of 41 isolates (69.5%) carried extended-spectrum β-lactamase (ESBL) genes, mainly *bla*_SHV-12_ and *bla*_CTX-M-65_, which mediate resistance to cephalosporins (Table 1).

**TABLE 1:** Selected antimicrobial resistance and virulence characteristics of 59 CRKP^*a*^

Characteristics	ST11 (*n* = 39)	ST15 (*n* = 12)	ST23 (*n* = 2)	ST147 (*n* = 2)	ST17 (*n* = 1)	ST218 (*n* = 1)	ST307 (*n* = 1)	ST437 (*n* = 1)	Total (*n* = 59)
Carbapenemase genes									
*bla*_KPC-2_	39 (100.0)	−	−	−	−	−	−	−	39 (66.1)
*bla*_NDM-5_	−	11 (91.7)	−	−	−	−	−	−	11 (18.6)
*bla*_NDM-1_	−	−	2 (100.0)	2 (100.0)	1 (100.0)	1 (100.0)	−	1 (100.0)	7 (11.9)
*bla*_IMP-4_	−	−	−	−	−	−	1 (100.0)	−	1 (1.7)
Negative	−	1 (8.3)	−	−	−	−	−	−	1 (1.7)
Extended-spectrum β-lactamase genes									
*bla*_CTX-M-65_	17 (43.6)	−	−	−	−	−	−	−	17 (28.8)
*bla*_SHV-12_	18 (46.2)	1 (8.3)	−	−	−	−	−	−	19 (32.2)
*bla*_CTX-M-15_	−	−	−	−	−	−	1 (100.0)	1 (100.0)	2 (3.4)
*bla*_CTX-M-15_; *bla*_SHV-12_	−	−	−	2 (100.0)	−	−	−	−	2 (3.4)
*bla*_CTX-M-27_; *bla*_SHV-12_	1 (25.6)	−	−	−	−	−	−	−	1 (1.7)
Negative	3 (7.7)	11 (91.7)	2 (100.0)	−	1 (100.0)	1 (100.0)	−	−	18 (30.5)
Capsule locus									
KL64	37 (94.9)			2 100.0)					39 (66.1)
KL47	2 (5.1)	−	−	−	−	−	−	−	2 (3.4)
KL48	−	11 (91.7)	−	−	−	−	−	−	11 (18.6)
KL24	−	1 (8.3)	−	−	−	−	−	−	1 (1.7)
KL1	−	−	2 (100.0)	−	−	−	−	−	2 (3.4)
KL57	−	−	−	−	−	1 (100.0)	−	−	1 (1.7)
KL112	−	−	−	−	1 (100.0)	−	−	−	1 (1.7)
KL402	−	−	−	−		−	1 (100.0)	−	1 (1.7)
KL36	−	−	−	−		−		1 (100.0)	1 (1.7)
Siderophore systems									
Yersiniabactin (*ybt*)	37 (94.9)	1 (8.3)	2 (100.0)	2 (100.0)	−	1 (100.0)	−	−	43 (72.9)
Negative	2 (5.1)	11 (91.7)	−	−	1 (100.0)	−	1 (100.0)	1 (100.0)	16 (27.1)
Aerobactin (*iuc1*)	36 (92.3)	−	2 (100.0)	−		1 (100.0)			39 (66.1)
Negative	3 (7.7)	12 (100.0)	−	2 (100.0)	1 (100.0)	−	1 (100.0)	1 (100.0)	20 (33.9)
Salmochelin (*iro1*)	−	−	2 (100.0)	−		1 (100.0)			3 (5.1)
Negative	39 (100.0)	12 (100.0)	−	2 (100.0)	1 (100.0)	−	1 (100.0)	1 (100.0)	56 (94.9)
Colibactin (*clb1*)	−	−	2 (100.0)	−	−	−			2 (3.4)
Negative	39 (100.0)	12 (100.0)	−	2 (100.0)	1 (100.0)	1 (100.0)	1 (100.0)	1 (100.0)	57 (96.61)
Hypermucoid locus (*RmpADC*)									
*rmp1-KpVp1*	−	−	2 (100.0)	−	−	1 (100.0)	−	−	3 (5.1)
*rmpA2*	36 (92.3)	−	2 (100.0)	−	−	1 (100.0)	−	−	39 (66.1)
Negative	3 (7.7)	12 (100.0)	−	2 (100.0)	1 (100.0)	−	1 (100.0)	1 (100.0)	20 (33.9)

^*a*^ “−” means negative.

**Fig 2:**
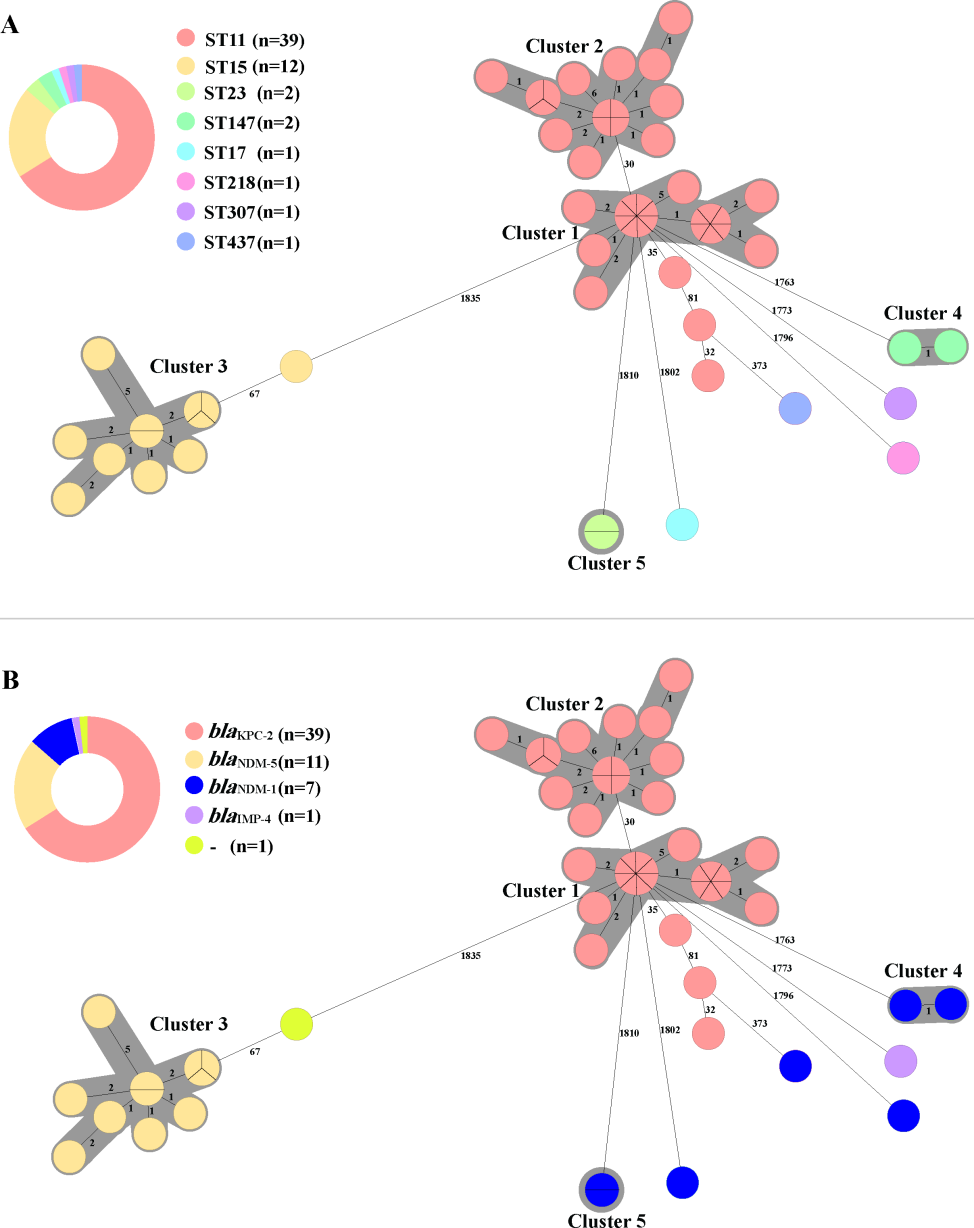
Comparison of sequence types and carbapenemase genes of 59 CRKP based on cgMLST. (**A**) The phylogenetic tree and sequence types distribution of 59 CRKP based on the core genome of *K. pneumoniae*. (**B**) The phylogenetic analysis of 59 CRKP isolates, derived from the core genome of *K. pneumoniae*, and their carbapenemase genes carriage profile. Each sphere-like node represents an isolate. If this sphere-like node is divided into several parts, it means that there are several isolates at this node, and the core genome of the isolates in the same sphere-like node are exactly the same. The number on the horizontal line between nodes indicates the number of core alleles that differ between two isolates, and the isolate that differs by ≤7 core alleles is a cluster, and each cluster is indicated by the gray-shaded area. “-” means negative.

Phylogenetic construction based on cgMLST revealed that 59 CRKP strains were divided into five clusters of homologous clones (Fig. 2A). Clusters 1 (21/59) and 2 (16/59) belonged to ST11. Cluster 3 included 11 isolates (11/59) of ST15. Clusters 4 (2/59) and 5 (2/59) belonged to ST147 and ST23, respectively. The analysis revealed that clonal dissemination was observed in 59 CRKP isolates in this study, including large clone clusters such as those within ST11 and ST15 and small clone clusters within ST23 and ST147 (Fig. 2A). We linked carbapenemase genes with STs via cgMLST and analyzed their spread (Fig. 2). We found that *bla*_KPC-2_ was spread mainly in CRKP through the clonal dissemination of ST11 and that the spread of *bla*_NDM-5_ was due to the ST15 clone.

We screened the virulence factors of the CRKP strains, including their capsular polysaccharide serotype, siderophore systems, colibactin, and hypermucoid locus, on the basis of their genome sequences (Table 1). Among the nine capsule serotypes, most of the CRKP strains expressed KL64 (66.1%, 39/59) and KL48 (18.7%, 11/59), and only a small number of CRKP strains expressed the remaining six KLs. We detected two isolates of ST23-KL1, a common type of hvKP, that carry yersiniabactin, aerobactin, salmochelin, and colibactin.

### Characterization of *bla*_NDM_-harboring plasmids

**Fig 3:**
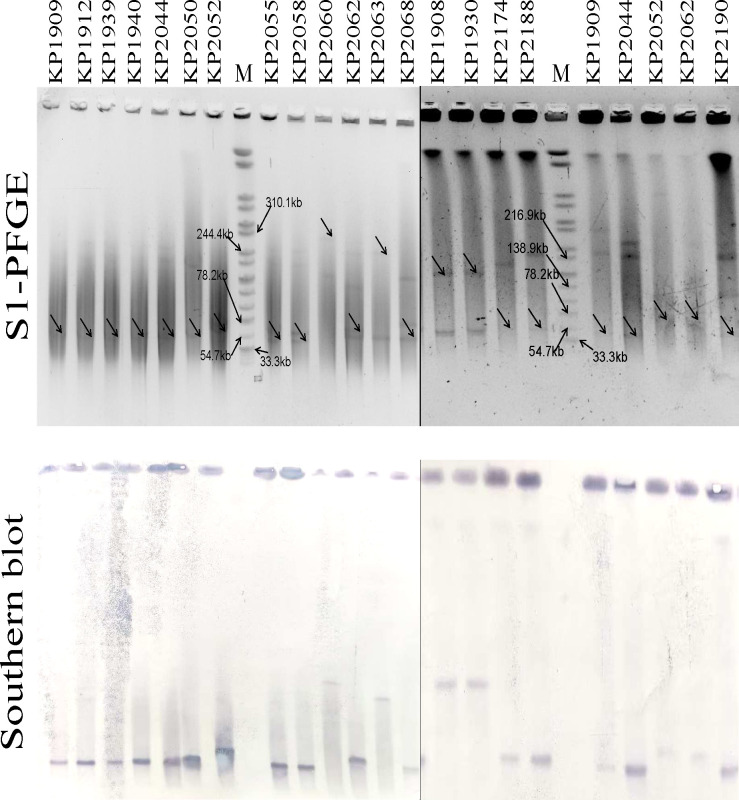
PFGE and Southern blot of 18 NDM-producing CRKP. “M” denotes the molecular marker of *Salmonella* serotype Braenderup strain H9812.

**Fig 4:**
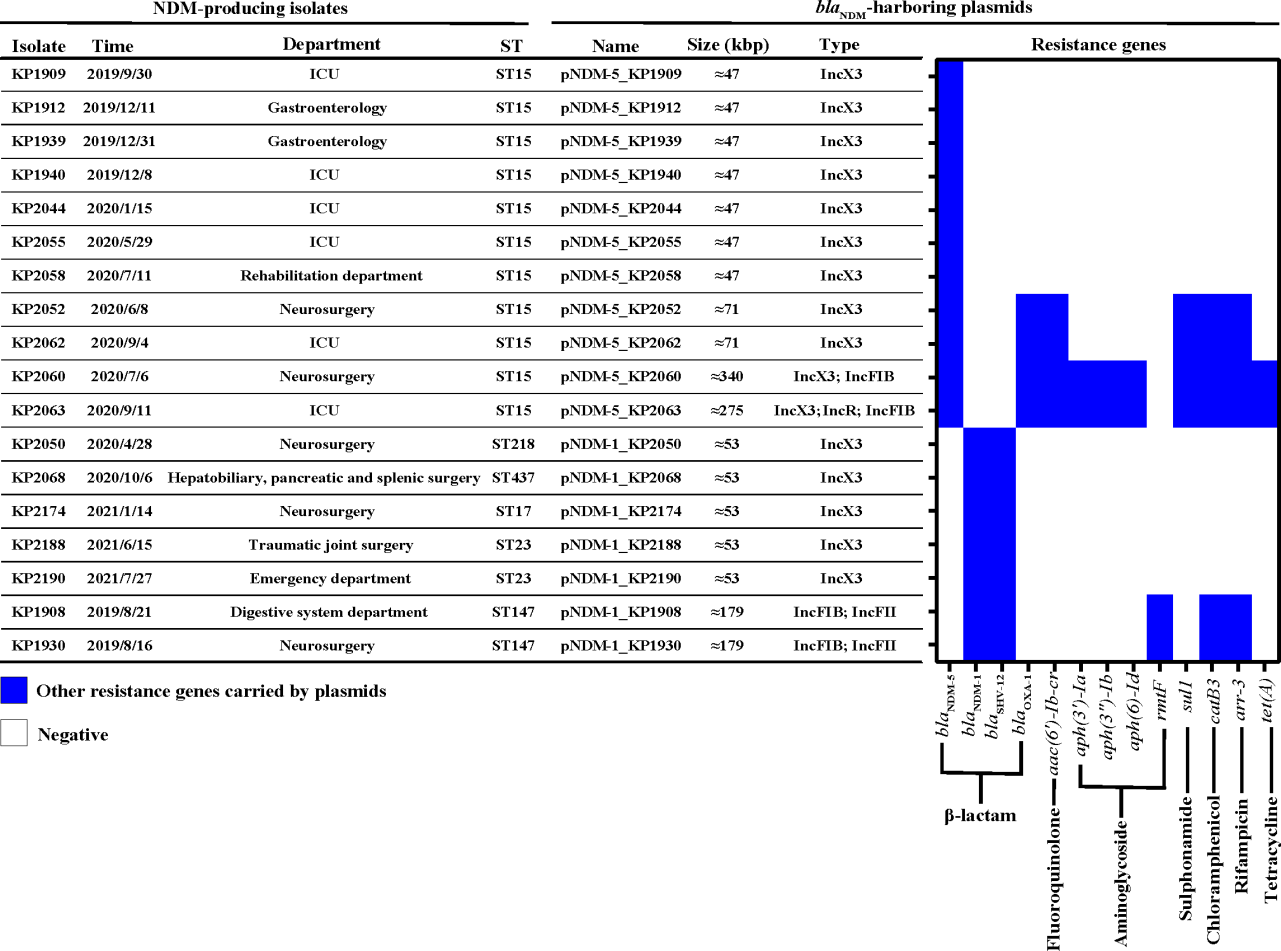
Characteristics of NDM-producing CRKP and *bla*_NDM_-harboring plasmids.

On the basis of the complete genome sequences assembled from the Nanopore MinION data, combined with the results of S1-PFGE and Southern blotting (Fig. 3), we analyzed the characteristics of the *bla*_NDM_-harboring plasmids in the CRKP strains (Fig. 4). *bla*_NDM_ is located on plasmids of six different sizes, of which approximately 47 kbp (7/18) and 53 kbp (5/18) are the most common. The plasmid incompatibility type was IncX3 (14/18), which included strains in five STs. These findings suggest that *bla*_NDM_ is likely to spread through the horizontal transfer of plasmids. Four *bla*_NDM_-harboring plasmids contained two or more plasmid incompatibility types, suggesting that plasmid fusion occurred. Among the 18 NDM-harboring plasmids, 11 were found to harbor additional resistance genes (11/18), with the highest count reaching nine different genes. This genetic diversity has the potential to confer resistance to strains across seven distinct classes of antibiotics. Furthermore, the dissemination of these NDM plasmids has led to infections across various hospital wards, notably infecting patients in the ICU (6/18) and neurosurgery (5/18).

### Comparative analysis of *bla*_NDM_-harboring plasmid sequences

**Fig 5:**
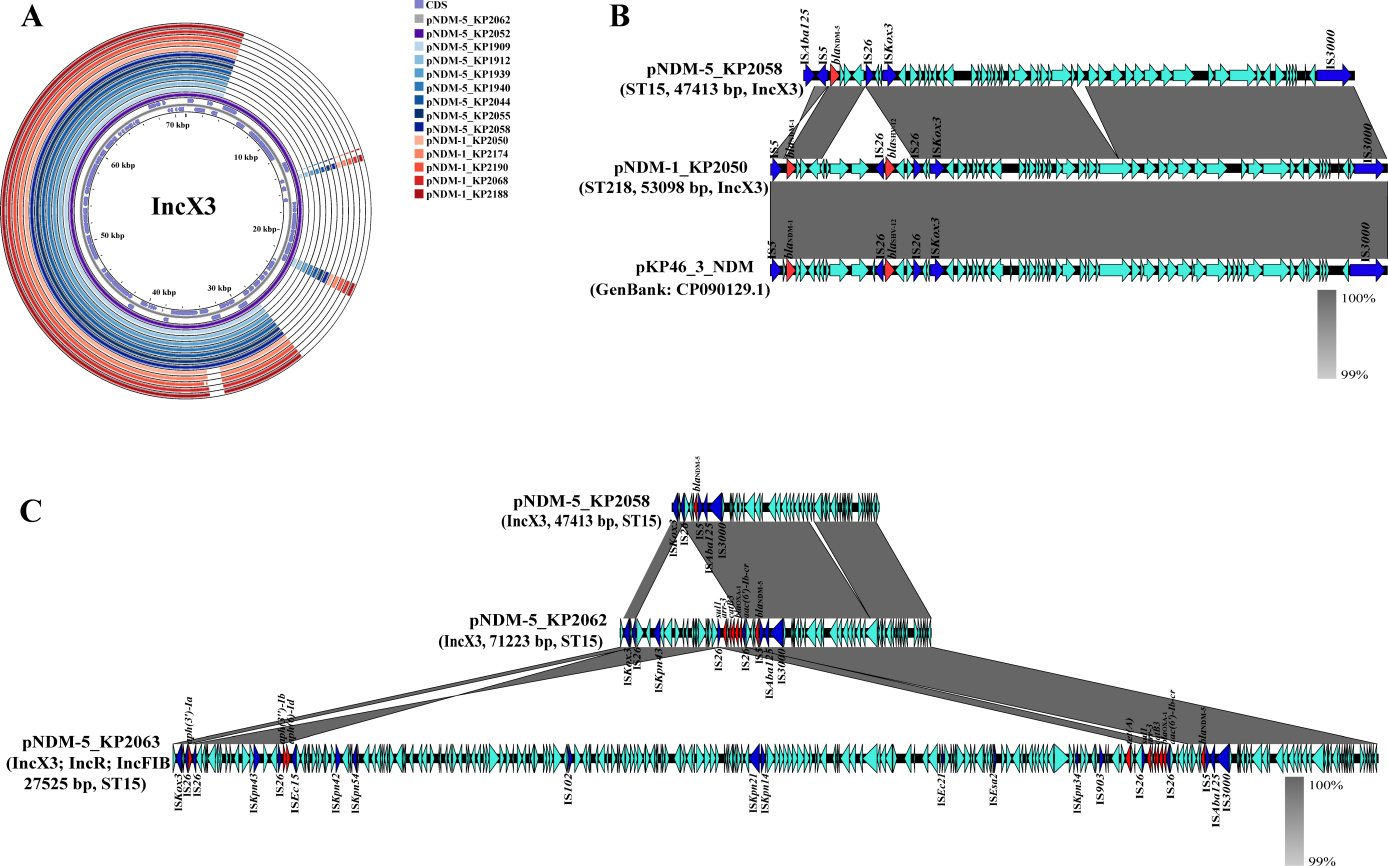
Comparison of sequences of *bla*_NDM_-harboring plasmids. (**A**) Comparison of the plasmid sequence of IncX3. The images were generated using Proksee online tool (https://proksee.ca). Using pNDM-5_KP2062 (71,223 bp) as a reference, comparison of sequences was performed on pNDM-5_KP2052, pNDM-5_KP1909, pNDM-5_KP1912, pNDM-5_KP1939, pNDM-5_KP1940, pNDM-5_KP2044, pNDM-5_KP2055, pNDM-5_KP2058, pNDM-1_KP2050, pNDM-1_KP2174, pNDM-1_KP2190, pNDM-1_KP2068, and pNDM-1_KP2188, which showed high sequence similarity to pNDM-5_KP2062. (**B**) Scaled, linear sequence comparison of *bla*_NDM-5_-harboring plasmid (pNDM-5_KP2058) with *bla*_NDM-1_-harboring plasmid (pNDM-1_KP2050) and plasmid pKP46_3_NDM (CP090129.1). (**C**) Scaled, linear sequence comparison of three *bla*_NDM-5_-harboring plasmids (pNDM-5_KP2058, pNDM-5_KP2062, pNDM-5_KP2063) from ST15 CRKP. Red indicates resistance genes and blue indicates insertion sequence-related genes. The shadow indicates that the similarity of the corresponding part is ≥99%.

To investigate whether *bla*_NDM_ is transmitted between strains via plasmids, we compared the sequences of *bla*_NDM_-harboring plasmids (Fig. 5). The results revealed that the sequence skeletons of the 14 IncX3 plasmids were highly similar (Fig. 5A). *bla*_NDM_ was disseminated by IncX3 plasmids to five different STs (ST15 [11/14], ST23 [2/14], ST218 [1/14], and ST17 [1/14]), and after *bla*_NDM_-harboring plasmids were disseminated to ST15, the ST15 CRKP strain producing NDM-5 grew rapidly. We also compared and analyzed the structure and sequence of the *bla*_NDM-5_- and *bla*_NDM-1_-harboring plasmids (Fig. 5B). Compared with pNDM-5_KP2058, an IS*26*-mediated sequence was inserted into the *bla*_NDM-1_ region of the plasmid pNDM-1_KP2058, and IS*26*-mediated gene recombination occurred, which transformed the original *bla*_NDM-5_ sequence on the plasmid into the *bla*_NDM-1_ sequence. Moreover, the sequence may have been reassembled from other *bla*_NDM-1_-harboring plasmids, such as the plasmid pKP46_3_NDM. In addition, the recombination event also introduced the ESBL gene *bla*_SHV-12_ into the plasmid. We found that during the process of dissemination, smaller plasmids could form larger plasmids through sequence insertion and plasmid integration and thus obtain other drug resistance genes. For example, the plasmid pNDM-5_KP2058 acquired IS*26*-mediated sequence integration, transforming it into pNDM-5_KP2062, and this insertion segment carried five resistance genes. Moreover, pNDM-5_KP2063 is composed of three different plasmid incompatibility types (IncX3, IncR, and IncFIB), and four resistance genes were obtained via plasmid integration (Fig. 5C).

### Transmissibility and fitness costs of *bla*_NDM_-harboring plasmids

To elucidate the transmissibility of *bla*_NDM_-harboring plasmids, we performed a comprehensive conjugation assay involving 18 NDM-producing CRKP strains. The results demonstrated that each of the *bla*_NDM_-harboring plasmids has the capacity for conjugative transfer, enabling the horizontal dissemination of resistance genes (Table 2).

**TABLE 2:** Antimicrobial susceptibility of 18 NDM-producing CRKP and their conjugates^*a*^

Isolate and conjugate	MIC (μg/mL)					
	MEM	IPM	ETP	CXM	CAZ	CTX
KP1908	128	32	128	>512	>256	>128
EC600-pNDM-1_KP1908	32	4	32	>512	>256	32
KP1909	256	64	256	>512	>256	>128
EC600-pNDM-5_KP1909	64	8	64	>512	>256	128
KP1912	128	32	256	>512	>256	>128
EC600-pNDM-5_KP1912	64	8	64	>512	>256	32
KP1930	128	16	256	>512	>256	>128
J53-pNDM-1_KP1930	32	4	64	>512	>256	32
KP1939	128	32	256	>512	>256	>128
EC600-pNDM-5_KP1939	64	8	64	>512	>256	32
KP1940	256	64	256	>512	>256	>128
EC600-pNDM-5_KP1940	64	8	64	>512	>256	128
KP2044	512	512	>512	>512	>256	>128
EC600-pNDM-5_KP2044	64	4	64	>512	>256	64
KP2050	128	16	64	>512	>256	>128
EC600-pNDM-1_KP2050	32	8	64	>512	>256	64
KP2052	128	32	256	>512	>256	>128
EC600-pNDM-5_KP2052	64	8	128	>512	>256	32
KP2055	256	32	256	>512	>256	>128
EC600-pNDM-5_KP2055	64	8	64	>512	>256	64
KP2058	256	16	256	>512	>256	>128
J53-pNDM-5_KP2058	64	8	128	>512	>256	128
KP2060	128	32	128	>512	>256	>128
EC600-pNDM-5_KP2060	64	8	64	>512	>256	32
KP2062	128	32	256	>512	>256	>128
EC600-pNDM-5_KP2062	64	8	64	>512	>256	32
KP2063	128	32	256	>512	>256	>128
EC600-pNDM-5_KP2063	64	8	64	>512	>256	32
KP2068	128	32	128	>512	>256	128
EC600-pNDM-1_KP2068	32	8	64	>512	>256	64
KP2174	64	16	64	>512	>256	128
EC600-pNDM-1_KP2174	32	8	64	>512	>256	32
KP2188	64	16	64	>512	>256	128
EC600-pNDM-1_KP2188	32	8	64	>512	>256	64
KP2190	128	16	128	>512	>256	128
EC600-pNDM-1_KP2190	64	8	64	>512	>256	64
EC600	0.03	0.5	0.016	128	0.25	0.06
J53	0.06	0.25	0.03	4	0.5	0.06

^*a*^ MEM, meropenem; IPM, imipenem; ETP, ertapenem; CXM, cefuroxime; CAZ, ceftazidime; CTX, cefotaxime. Donor strain: KP1908, KP1909, KP1912, KP1930, KP1939, KP1940, KP2044, KP2050, KP2052, KP2055, KP2058, KP2060, KP2062, KP2063, KP2068, KP2174, KP2188, KP2190; conjugate: EC600-pNDM-1_KP1908, EC600-pNDM-5_KP1909, EC600-pNDM-5_KP1912, J53-pNDM-1_KP1930, EC600-pNDM-5_KP1939, EC600-pNDM-5_KP1940, EC600-pNDM-5_KP2044, EC600-pNDM-1_KP2050, EC600-pNDM-5_KP2052, EC600-pNDM-5_KP2055, J53-pNDM-5_KP2058, EC600-pNDM-5_KP2060, EC600-pNDM-5_KP2062, EC600-pNDM-5_KP2063, EC600-pNDM-1_KP2068, EC600-pNDM-1_KP2174, EC600-pNDM-1_KP2188, EC600-pNDM-1_KP2190; recipient strain: EC600, J53.

**Fig 6:**
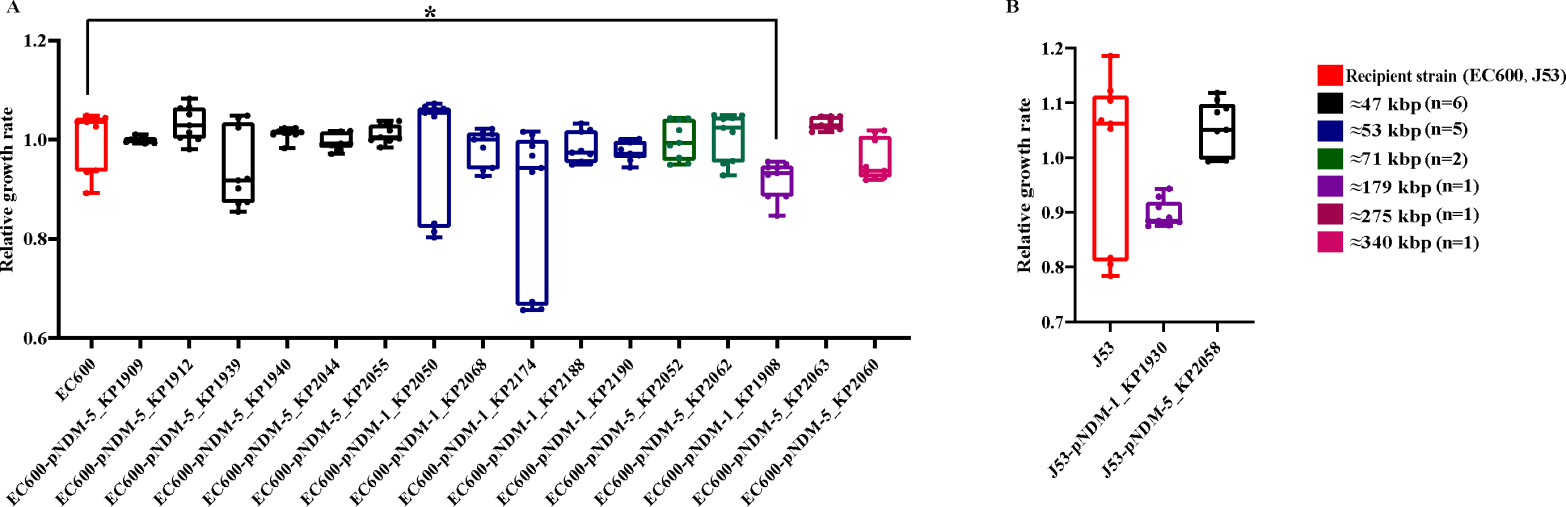
Relative growth rates of 18 NDM-producing CRKP and their conjugates. (**A**) Relative growth rate of 16 conjugates with EC600 as recipient bacteria. (**B**) Relative growth rate of 16 conjugates with J53 as recipient bacteria. Different colors represent *bla*_NDM_-harboring conjugates of different sizes. “*” means *P* < 0.05, and other conjugates which are not marked with statistical difference indicate that there is no significant difference in growth rate between them and the recipient bacteria. Conjugate: EC600-pNDM-1_KP1908, EC600-pNDM-5_KP1909, EC600-pNDM-5_KP1912, J53-pNDM-1_KP1930, EC600-pNDM-5_KP1939, EC600-pNDM-5_KP1940, EC600-pNDM-5_KP2044, EC600-pNDM-1_KP2050, EC600-pNDM-5_KP2052, EC600-pNDM-5_KP2055, J53-pNDM-5_KP2058, EC600-pNDM-5_KP2060, EC600-pNDM-5_KP2062, EC600-pNDM-5_KP2063, EC600-pNDM-1_KP2068, EC600-pNDM-1_KP2174, EC600-pNDM-1_KP2188, EC600-pNDM-1_KP2190.

To investigate the adaptability of *bla*_NDM_-harboring plasmids, we conducted a comparative analysis of the relative growth rates of the recipient and donor strains (Fig. 6). Our findings revealed that the vast majority of these plasmids (17/18) did not incur any adaptive costs posthorizontal gene transfer to the recipient strains.

Furthermore, our plasmid stability assays indicated that *bla*_NDM_-harboring plasmids retention rate reached 100%. These plasmids exhibit remarkable stability after passage, maintaining their integrity even in the absence of antibiotic selection pressure. Intriguingly, once the other bacterial strains acquired the *bla*_NDM_-harboring plasmid, the plasmids demonstrated robust resistance to loss or inactivation during subsequent passages.

### Virulence analysis of NDM-producing CRKP strains

In this study, CR-hvKP was defined as a CRKP isolate displaying a biomarker with hvKP characteristics (*iuc + rmpA*) or a CRKP isolate with a toxicity level significantly greater than that of the low-virulence strain in the *G. mellonella* larvae infection model.

NDM-producing strains carry many virulence factors. We screened two CR-hvKP clones, ST23 and ST218, through virulence factors, both of which carry yersiniabactin, aerobactin, salmochelin, the hypermucoid gene, and colibactin, highlighting their potential for causing severe infections (Table 3).

**TABLE 3:** Molecular characterization associated with the virulence of NDM-producing CRKP^*a*^

Virulence factors	ST15 (*n* = 11)	ST23 (*n* = 2)	ST147 (*n* = 2)	ST218 (*n* = 1)	ST17 (*n* = 1)	ST437 (*n* = 1)
Capsule locus						
KL64	−	−	2 (100.0)	−	−	−
KL48	11 (100.0)	−	−	−	−	−
KL1	−	2 (100.0)	−	−	−	−
KL57	−	−	−	1 (100.0)	−	−
KL112	−	−	−	−	1 (100.0)	−
KL36	−	−	−	−	−	1 (100.0)
Siderophore systems						
Yersiniabactin						
*ybt*9-ICEKp3	−	−	−	1 (100.0)	−	−
*ybt*9-ICEKp3 (truncated)		−	−	−	−	−
*ybt*1-ICEKp10	−	2 (100.0)	−	−	−	−
*ybt*16-ICEKp12	−	−	2 (100.0)	−	−	−
Aerobactin					−	−
*iuc1*	−	2 (100.0)	−	1 (100.0)		
Salmochelin					−	−
*iro1*	−	2 (100.0)	−	1 (100.0)	−	−
Hypermucoid locus (*RmpADC*)						
*rmp1-KpVp1*	−	2 (100.0)	−	1 (100.0)	−	−
*rmpA2*	−	2 (100.0)	−	1 (100.0)	−	−
Colibactin						
*clb1*	−	2 (100.0)	−	−	−	−

^*a*^ “−” means negative.

**Fig 7:**
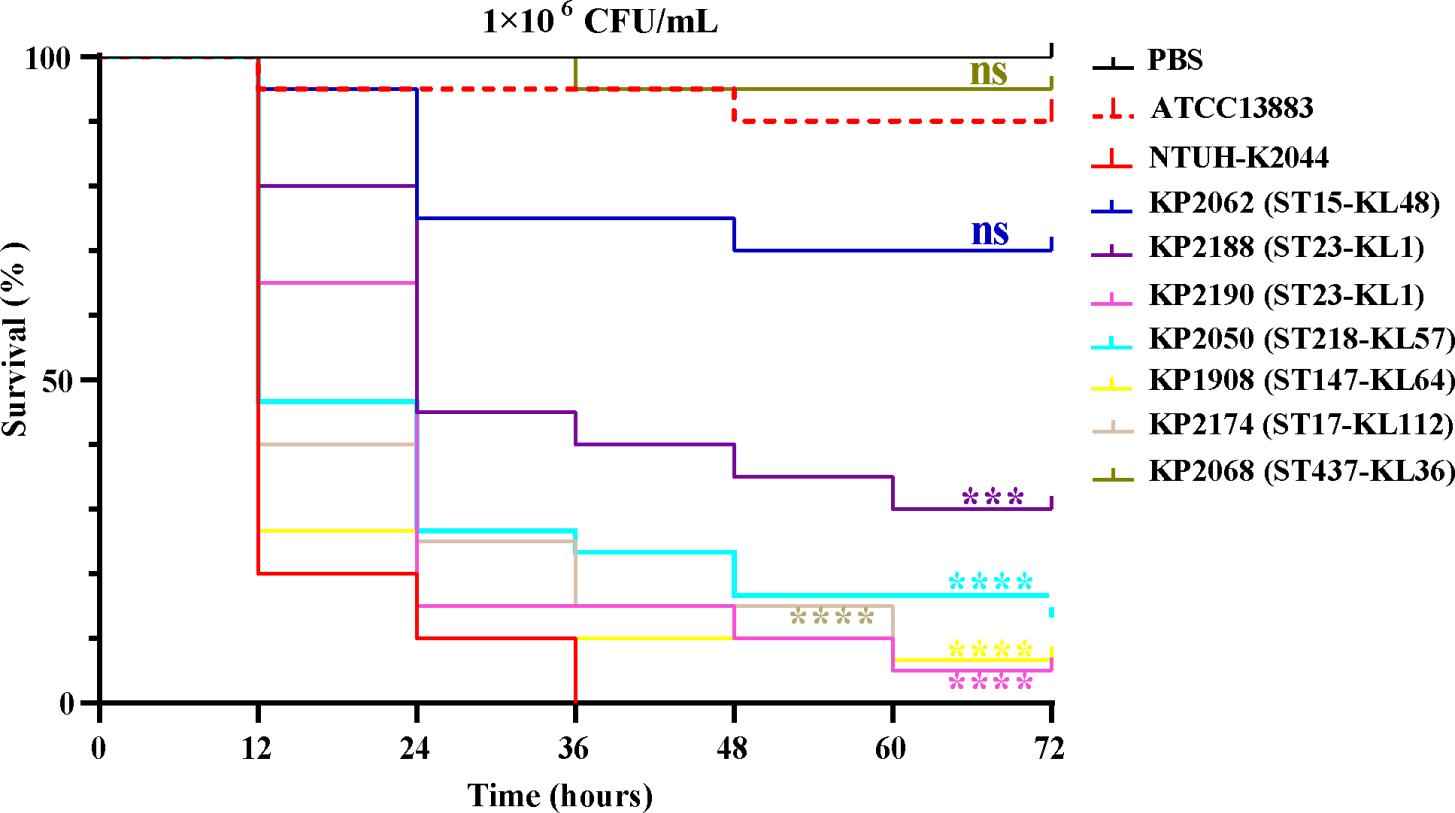
The *In vivo* virulence of NDM-producing in *G. mellonella* model. ATCC13883 and NTUH-K2044 are low-virulence control strains and high-virulence control strains, respectively. “ns” indicates that there is no significant difference compared with ATCC13883; “*” means *P* < 0.05 compared with ATCC13883; “*” means *P* < 0.001 compared with ATCC13883; “*” means *P* < 0.0001 compared with ATCC13883.

Seven representative strains with different KLs were selected for virulence evaluation in the *G. mellonella* larvae infection model (Fig. 7). The virulence of ST23-KL1 (*P* < 0.001 [KP2188], *P* < 0.0001 [KP2190]), ST218-KL57 (*P* < 0.0001), and ST17-KL112 (*P* < 0.0001) and that of ST147-KL64 (*P* < 0.0001) was significantly greater than that of ATCC13883, and both strains harbored *bla*_NDM-1_ plasmids, indicating that these were CR-hvKP strains with high virulence and high drug resistance.

## DISCUSSION

In China, CRKP is a critical threat to public health, especially ST11 strains, which produce KPC-2. The proportion of CRKP strains that produces NDM is relatively small. In this study, we describe the molecular features of CRKP strains isolated from patients receiving care at a tertiary hospital in China from 2019 to 2021, and identify their antimicrobial resistance and molecular epidemiological characteristics. Notably, there was a high proportion of NDM-producing CRKP strains (18/59) in this study, in which the NDM-producing ST15 (11/18) clone spread widely in the hospital and six isolates were NDM-producing CR-hvKP (6/18). Moreover, we found that the dissemination of *bla*_NDM_-harboring plasmids was closely related to these events.

CRKP exhibits a multidrug resistance phenotype; however, all CRKP strains are not resistant to colistin or tigecycline. In a previous study in China, the resistance rate of CRKP to tigecycline was 27.5% (5), but the resistance rates to tigecycline and colistin in this study were much lower. However, the use of tigecycline to treat infections remains controversial due to its low plasma concentrations. Although colistin is the last line of defense in the treatment of CRKP infection, there have been reports of colistin-resistant CRKP strains (29), and its potential neurotoxicity and ototoxicity suggest that colistin should still be used cautiously in the clinical treatment of CRKP. The results of this study revealed that the production of carbapenemase was the main mechanism leading to carbapenem resistance in CRKP isolates. Research has shown that ceftazidime/avibactam is a suitable clinical choice for treating CRKP infections, especially those caused by KPC-producing CRKP (1), but we found it to be ineffective against CRKP strains that produce metallo-β-lactamases (especially NDM). The acquisition of metalloenzymes in different clones, as well as the emergence of potential resistance to ceftazidime-avibactam due to mutations or high expression of KPC (5), suggests that we should continue to monitor antimicrobial resistance trends and use antimicrobials according to enzyme types present.

In this study, we found that *bla*_KPC-2_ is transmitted by the ST11 clone and that ST11- *bla*_KPC-2_ is the main genotype of CRKP, which is similar to the findings of most previous studies (5, 6). Unlike the prevalence of KPC-producing CRKP strains, NDM-producing CRKP strains has a high diversity of STs, and high-risk clones producing NDM are relatively rare (8–10). Notably, we found a high number of NDM-5-producing ST15-KL48 CRKP clones, which is completely different from the findings of other reports. A study on the epidemiology of CRKP in Zhejiang Province (2008–2018) revealed that NDM-producing isolates accounted for only 2.8% of CRKP strains, and most of them were ST11 strains (30). Most studies have shown that KPC is dominant in ST15 CRKP (30–32), but in our research, NDM was the main type of carbapenemase in ST15. The ST15 strains are classic multidrug-resistant *K. pneumoniae* strains (33), with reports indicating that ST15 can cause bacteremia in adults (5). Compared with the predominant KPC strains, the ST15 strains developed resistance to ceftazidime-avibactam due to the production of NDM. Moreover, studies have shown that ST15 CRKP strains producing NDM-1 have acquired virulence plasmids, thereby increasing the virulence of the strains (14). Various studies have indicated that ST15 clones carrying NDM exhibit a trend of high-level resistance and virulence convergence, which has prompted us to focus on this high-risk clone.

Although *bla*_NDM_ is encoded in the genome in a few cases, its widespread dissemination in *Enterobacteriaceae* is primarily mediated by certain plasmids, especially the IncX3 type (22). We detected the *bla*_NDM_-harboring plasmid in 16 (16/18) CRKP strains from patients in multiple hospital wards, and the analysis indicated that the spread of *bla*_NDM_ in this study was inextricably linked to the IncX3 plasmid. Moreover, the presence of *bla*_NDM_ on the IncFIB, IncFII, and IncR plasmids also suggests that the acquisition of *bla*_NDM_ by different plasmids is a common phenomenon, as observed in other studies (9, 14, 20, 34). In this study, *bla*_NDM_ was disseminated by IncX3 plasmids to five different STs, and after *bla*_NDM_-harboring plasmids were disseminated to ST15, there was an outbreak of NDM-producing ST15 CRKP strains. Moreover, we found that the *bla*_NDM_-harboring plasmid has advantages in terms of dissemination, including horizontal transfer ability, suitable adaptability, and stability. They also undergo evolution that favors drug resistance during the transmission process. These characteristics suggest that the *bla*_NDM_-harboring plasmid can rapidly spread and persist over a large area in medical institutions, which is highly detrimental to the control of NDM-producing CRKP.

At present, there is no unified standard for the identification of hvKP. Studies have shown that the use of high virulence-related factors as the criterion for determining hvKP is not comprehensive (35); therefore, we selected a scheme that combines virulence genes with the *G. mellonella* larvae infection model in this study, and CRKP strains with hvKP characteristic biomarkers (*iuc* + hypermucoid-related gene *rmpA*) or virulence levels significantly higher than those of the low-virulence control strain were defined as CR-hvKP (5, 35). To date, most CR-hvKP isolates produce KPC-2, especially in China, while NDM-producing CR-hvKP strains have been reported relatively rarely (13, 23), and the number of cloning transmission events is even lower. However, we found that there is emerging high-risk clone transmission of ST23-KL1 CR-hvKP producing NDM-1 in our medical institutions, which needs special attention. ST23 CR-hvKP was first reported in Russia in 2018, and its drug-resistant plasmid carries both the carbapenemase gene (*bla*_OXA-48_) and the ESBL gene (*bla*_CTX-M-15_). Studies have shown that hvKP evolved into CR-hvKP when drug-resistant plasmids were obtained (36). In this study, *bla*_NDM_-harboring plasmids were disseminated in all six clones, including ST23, which is a common clone of hvKP. It is speculated that the carbapenem resistance of the ST23 CR-hvKP strains in this study was acquired by obtaining *bla*_NDM_-IncX3 plasmids. The dissemination of *bla*_NDM_-harboring plasmids makes the formation of highly virulent and drug-resistant strains easier. Therefore, it is not only necessary for the detection of drug resistance genes but also important to further study intervention measures to eliminate the *bla*_NDM_-IncX3 plasmid. In this study, NDM-producing CR-hvKP strains exhibited a certain degree of polymorphism. In addition to the most common ST23-KL1, less frequently encountered types, such as ST147-KL64, ST218-KL57, and ST17-KL112, were also detected (37), indicating that CR-hvKP has mutated to include more STs and serotypes. The transfer and integration of *bla*_NDM_-IncX3 plasmids have increased the mutation and dissemination rates of CR-hvKP, affecting not only the common STs/KLs of hvKP but also an increasing number of rare STs/KL types of CR-hvKP, which has serious consequences for infected patients.

### Conclusion

Plasmids play crucial roles in the dissemination of *bla*_NDM_, especially *bla*_NDM_ harbored in the IncX3 plasmid. The transmissibility of *bla*_NDM_-harboring plasmids has dangerous implications for CRKP infection: when the *bla*_NDM_-harboring plasmid disseminates to ST15, it causes an outbreak of NDM-producing ST15 CRKP, and the prevalence of high-resistance clones makes the high-resistance phenotype spread more quickly and widely. When the *bla*_NDM_-harboring plasmid disseminates to a highly virulent clone (such as ST23), the highly virulent clone obtains a high-resistance phenotype and further evolves into CR-hvKP. The dissemination of *bla*_NDM_-harboring plasmids has caused great harm to hospitalized patients, so it is necessary to formulate strict measures to prevent and control the development of *bla*_NDM_-harboring plasmids.

## Data Availability

Genomic data for the isolates sequenced in this study have been deposited in the NCBI with BioProject and BioSamples numbers PRJNA1181777 and SAMN44662701-SAMN44662759, respectively.

## SUPPLEMENTAL MATERIAL

Table S1 spectrum.01968-24-s0001.docx Distribution of CRKP specimens.

Table S2 spectrum.01968-24-s0002.docx Clinical characteristics of CRKP.

ASM does not own the copyrights to Supplemental Material that may be linked to, or accessed through, an article. The authors have granted ASM a non-exclusive, world-wide license to publish the Supplemental Material files. Please contact the corresponding author directly for reuse.
