# Erratum for Gu et al., “Dissemination of *bla*_NDM_-harboring plasmids in carbapenem-resistant and hypervirulent *Klebsiella pneumoniae”*

**DOI:** 10.1128/spectrum.00577-25

**Published:** 2025-04-04

**Authors:** Yihai Gu, Xiao Wang, Wei Zhang, Rui Weng, Qiucheng Shi, Xuan Hou, Hui Wang, Minghui Deng, Jian Mou, Yan Jiang

## ERRATUM

Volume 13, no. 3, e01968-24, 2025, https://doi.org/10.1128/spectrum.01968-24. Page 14, Author Affiliations: The first affiliation should read "Department of Microbiology, 3201 Hospital, School of Medicine, Xi'an Jiaotong University, Hanzhong, China.”

