# Supplementary material for: Dissemination of blaNDM-harboring plasmids in carbapenem-resistant and hypervirulent Klebsiella pneumoniae
Source: Microbiol Spectr. 2025 Feb 12;13(3):e01968-24. doi: 10.1128/spectrum.01968-24 (PMC11878072; doi:10.1128/spectrum.01968-24)
Supplement: Table S1 — Distribution of CRKP specimens. [file spectrum.01968-24-s0001.docx]

**Supplementary table 1** Distribution of CRKP specimens

| Specimen Type | *n* | % |
| --- | --- | --- |
| Respiratory | 24 | 40.7 |
| Blood | 14 | 23.7 |
| Urine | 7 | 11.9 |
| Secretion | 6 | 10.2 |
| Puncture fluid | 2 | 3.4 |
| Cerebrospinal fluid | 1 | 1.7 |
| Drainage | 1 | 1.7 |
| Pus | 1 | 1.7 |
| Hydrothorax | 1 | 1.7 |
| Ascites | 1 | 1.7 |
| Bone marrow | 1 | 1.7 |
| Total | 59 | 100 |
