# Supplementary material for: Dissemination of blaNDM-harboring plasmids in carbapenem-resistant and hypervirulent Klebsiella pneumoniae
Source: Microbiol Spectr. 2025 Feb 12;13(3):e01968-24. doi: 10.1128/spectrum.01968-24 (PMC11878072; doi:10.1128/spectrum.01968-24)
Supplement: Table S2 — Clinical characteristics of CRKP. [file spectrum.01968-24-s0002.docx]

**Supplementary table 2** Clinical characteristics of carbapenem-resistant *Klebsiella pneumoniae*

| Clinical characteristics of CRKP (*n*=59) | | *n* | % |
| --- | --- | --- | --- |
| Age ($\bar{x}$± *s*) | | 55.16 ± 19.79 | |
| Gender |  |  |  |
|  | Male | 34 | 57.6 |
|  | Female | 25 | 42.4 |
| Department |  |  |  |
|  | ICU | 18 | 30.5 |
|  | Neurosurgery | 12 | 20.3 |
|  | Department of respiratory and critical care medicine | 7 | 11.9 |
|  | Rehabilitation department | 5 | 8.5 |
|  | Hepatobiliary, pancreatic and splenic surgery | 4 | 6.8 |
|  | Gastroenterology | 2 | 3.4 |
|  | Emergency department | 2 | 3.4 |
|  | Digestive system department | 2 | 3.4 |
|  | Cardiothoracic surgery | 1 | 1.7 |
|  | Urological surgery | 1 | 1.7 |
|  | Traumatic joint surgery | 1 | 1.7 |
|  | Orthopedics department | 1 | 1.7 |
|  | Burn | 1 | 1.7 |
|  | Oncology department | 1 | 1.7 |
|  | Hematology | 1 | 1.7 |
